# South African healthcare workers’ knowledge of dolutegravir's drug–drug interactions in the first year of its rollout: a cross‐sectional online survey

**DOI:** 10.1002/jia2.25885

**Published:** 2022-03-07

**Authors:** Briony S. Chisholm, Annoesjka M. Swart, Marc Blockman

**Affiliations:** ^1^ Division of Clinical Pharmacology National HIV and TB Healthcare Worker Hotline Medicines Information Centre University of Cape Town Cape Town South Africa; ^2^ Division of Clinical Pharmacology University of Cape Town Cape Town South Africa

**Keywords:** health personnel, antiretroviral agents, drug interactions, knowledge, practice guideline, training

## Abstract

**Introduction:**

In December 2019, dolutegravir‐based treatment was recommended as first‐line antiretroviral therapy (ART) in South Africa. Dolutegravir has clinically significant interactions with several commonly used drugs, such as rifampicin, metformin and cation‐containing medicines. National guidelines detail these interactions and how to manage them. While previous international studies have shown low healthcare worker knowledge of drug–drug interactions, there is a paucity of information on antiretroviral interaction knowledge in the South African setting, where much ART is nurse‐led. The study aimed to determine this knowledge and to describe which variables were associated with gaps in knowledge.

**Methods:**

An anonymous online survey of healthcare workers in the field of HIV was conducted in August/September 2020. The survey was designed, tested and piloted, and included sections on demographics, guideline access and training, interaction knowledge, counselling and the effect of COVID‐19. Dissemination was via e‐mail and social media (convenience sampling). Descriptive and inferential analysis was done using proportions and the 95% confidence interval to determine relationships between independent and dependent variables. Research ethics approval was obtained from the University of Cape Town's Human Research Ethics Committee (HREC Ref: 357/2020).

**Results and discussion:**

In total, 1950 survey responses were included in the analysis – 47.1% nurses, 35.8% doctors and 8.9% pharmacists. When asked whether they were aware that dolutegravir has interactions, 70% said yes, 13.9% said no and 16.1% did not answer. Knowledge of specific interactions and the dosing changes needed was low with a wide range between different drugs: 79.7% knew to double the dolutegravir dose with rifampicin, but with calcium, 5.1% picked both correct dosing options and 33.7% picked one of the two correct options. Access to guidelines and training were positively associated with drug interaction knowledge.

**Conclusions:**

There are gaps in the awareness and knowledge of dolutegravir interactions and how to adjust dosing among South African healthcare workers.

## 1 INTRODUCTION

South Africa has the largest HIV treatment programme globally: 7.5 million people were living with HIV in 2019, 70% of them on antiretroviral therapy (ART) [1]. The South African national HIV guidelines were updated in December 2019 to recommend dolutegravir‐based ART as first‐line treatment [2], in line with World Health Organization recommendations [3]. Dolutegravir, an integrase strand transfer inhibitor (INSTI), has been shown to be safe, effective and well tolerated [4] with a shorter median time to viral suppression compared to other antiretroviral regimens [4, 5] and a high barrier to the development of drug resistance [6].

With South Africa's significant HIV burden and limited human resources, task‐shifting to nurse‐initiated and managed ART (NIMART) was introduced in 2010 [7]. Community health workers (CHWs) also form an integral part of the primary health team and their activities include providing health education and supporting ART adherence [8, 9, 10]. Most professional staff receive interaction training as part of their tertiary education but this would not have included training on dolutegravir, a new drug in the South African context. When updated guidelines are released, training of all healthcare workers (HCWs) is conducted by the Department of Health, various non‐governmental organizations and private trainers at a national and provincial level both online and face‐to‐face, often using the “train the trainer” method. CHWs and counsellors are included in this training in some districts.

While one of the touted advantages of dolutegravir is its lower potential for drug–drug interactions (DDIs) [11], pharmacokinetic studies have shown interactions with some commonly used drugs. These include cation‐containing medicines [12, 13], metformin [14], rifampicin [15, 16] and some anti‐epileptic drugs [17, 18, 19]. The clinical significance of these interactions is currently unclear with only a few case reports and one small rifampicin study providing data [18, 20, 21, 22, 23, 24, 25, 26]. Dolutegravir's lack of an interaction with oral contraceptives [27, 28] is a major advantage.

The DDIs require adjusted dosing and/or dosing schedules – clearly stated in the South African national ART guidelines [2] – to prevent adverse effects and loss of efficacy of dolutegravir. Failure to adjust correctly could result in the development of HIV‐1 resistance, treatment failure and HIV transmission.

Several antiretroviral prescription audits have been conducted in the African setting showing a prevalence of clinically relevant DDIs in 18.7–84% of patients [29, 30, 31]. These studies included non‐INSTI regimens, which may be more vulnerable to DDIs. While information on DDIs, the steps required to prevent them and the prevalence of prescribing errors due to DDIs is available, there is a paucity of information on the knowledge of HCWs regarding interactions, especially in the context of antiretroviral interactions, in the nursing profession and in the South African setting.

International studies of HCW knowledge of non‐antiretroviral DDIs have shown low levels of knowledge [32, 33, 34]. One small study of physicians in a United Kingdom‐based hospital showed that only 36% of clinically relevant interactions with ART were identified [35]. An American survey showed that 40% of resident and attending physicians and 90% of infectious disease/HIV specialists correctly answered case‐based DDI questions [36].

The primary aim of our study was to determine dolutegravir interaction knowledge of South African HCWs involved in HIV care and to describe which variables were associated with gaps in knowledge.

## 2 METHODS

### 2.1 Research design

We conducted a cross‐sectional, descriptive study using an anonymous online survey of HCWs in the field of HIV in South Africa. The study was conducted from the National HIV and TB Healthcare Worker Hotline based in the Division of Clinical Pharmacology at the University of Cape Town. The toll‐free hotline has been running since 2008 and answers around 500 HIV‐ and TB‐related clinical queries a month from HCWs across South Africa.

### 2.2 Population and sampling

The survey was disseminated to HCWs who had used the hotline and by relevant HIV‐focused organizations, such as the Southern African HIV Clinicians Society and TB HIV Care, via e‐mail, SMS and social media (convenience sampling [37]). It was conducted in English and was designed to exclude non‐consenting participants. It ran for eight weeks in August and September 2020.

### 2.3 Instrumentation

No similar published questionnaires were found, so the survey was designed on REDCap™, guided by *The SAGE Handbook of Survey Methodology* [37]. Input from experts and non‐experts was obtained to ensure reliability and validity – face validity (four lay people); content validity (five HIV experts, three social scientists and one lay person); and test‐retest for reliability (five hotline pharmacists). It was piloted by 10 HCWs in the field and minor changes made. Pilot responses were excluded from analysis.

The survey used branching logic and consisted of five sections: demographics; guideline access and training; interaction knowledge; HCW‐reported counselling on interactions; and the effect of COVID‐19.

### 2.4 Data analysis

Simple descriptive statistics were calculated on Excel™ and statistical analyses were performed using STATA™ software (StataCorp. 2019. Stata Statistical Software: Release 16. College Station, TX: StataCorp LLC). Descriptive statistics were used to describe demographic data, access to guidelines and training, and interaction awareness and knowledge.

Analysis was performed on completed (1350) and incomplete surveys (600), using the number of responses to each question as the denominator. As an example, when calculating the number of HCWs who knew the specific dosage/dosing changes, the denominator used was the number of people who saw the question (by branching logic, those who were not aware of an interaction with the drug did not see the question on dosing changes), so these proportions describe only the knowledge of those who knew of an interaction. Blank responses were excluded in the inferential analyses of differences in knowledge by variable.

In the inferential analysis, proportions were used to describe positive responses to each question and the 95% confidence interval (CI) was calculated to find statistical differences. All tests were two‐sided, and *p* ≤ 0.05 was considered statistically significant. To determine if relationships existed between independent and dependent variables, proportions tests were used (significance level 0.05). The number of variables to compare was large so, instead of performing individual proportions tests within variables, the 95% CI was chosen to perform the tests.

### 2.5 Ethical considerations

Research ethics approval was obtained from the University of Cape Town's Human Research Ethics Committee (HREC Ref: 357/2020).

## 3 RESULTS AND DISCUSSION

Due to the nature of the dissemination of the survey URL via multiple platforms, response rate calculation was not possible. Of 2549 surveys submitted, 599 were not suitable for analysis. Seventy‐nine percent of these were excluded because no responses were provided to, or beyond, demographic variables and 21% did not meet inclusion criteria. Statistical analyses were conducted on the remaining 1950 surveys.

### 3.1 Demographics

Most respondents were nurses (47.1%), doctors (35.8%) and pharmacists (8.9%), with a median number of years of HIV experience of 10 years (IQR 5–15 years). Respondents were well spread across the country by urban/rural spread and public/private sector ratio. Most respondents worked at primary health clinics (Table 1).

**Table 1:** Survey respondent demographics and training by profession

	Participants, *n* (%)
Training received by profession (*n*)	
Community health worker (*n* = 35)	8 (22.9)^a^
Counsellor (*n* = 47)	22 (46.8)^a^
Doctor (*n* = 699)	389 (55.7)^a^
Nurse (*n* = 918)	570 (62.1)^a^
Pharmacist (*n* = 173)	88 (50.9)^a^
Other healthcare worker (*n* = 73)	26 (35.6)^a^
Age, years	
Median [IQR]	41 [33–51]
18–27	132 (6.8)
28–37	657 (33.7)
38–47	529 (27.1)
48–57	377 (19.3)
> 58	244 (12.5)
Missing	11 (0.6)
HIV experience, years	
Median [IQR]	10 [5–15]
<1	42 (2.2)
1–5	578 (29.6)
6–10	591 (30.3)
> 10	739 (37.9)
Area	
Rural	800 (41.0)
Urban	1138 (58.4)
Missing	12 (0.6)
Sector	
Public	1512 (77.5)
Private	427 (21.9)
Missing	11 (0.6)
Facility	
Mobile clinic	24 (1.2)
Satellite clinic	15 (0.8)
Primary health clinic	659 (33.8)
Community health centre	276 (14.2)
District hospital	277 (14.2)
Regional/tertiary/specialized hospital	269 (13.8)
Private practice	168 (8.6)
Private hospital	65 (3.3)
Other	189 (9.7)
Missing	8 (0.4)

Abbreviation: IQR, interquartile range.

^a^Percentages by profession category.

### 3.2 Awareness of dolutegravir interactions

Including all surveys analysed (1950), 70% of all HCWs answered “yes” to the question “Are you aware that dolutegravir interacts with some other medications?”; 13.9% responded “no”; and 16.1% had dropped out of the survey by this point. When excluding the 314 participants who had dropped out by/in this section, 83.4% responded yes. Of those who were aware that dolutegravir has interactions, between 53.5% and 61.5% were aware of the interaction with cations; 86.9% with rifampicin and 78.2% with metformin. With the antiepileptics, proportions of respondents aware of the interactions with carbamazepine, phenobarbitone and phenytoin were 58.7%, 44.4% and 50.1%, respectively. Looking at drugs that do not interact with dolutegravir, 17.6% thought there was an interaction with oral contraceptives (Table 2).

**Table 2:** Awareness that dolutegravir has interactions and of specific drugs

	Participants, *n* (%)	Participants, *n* (%) including all
Awareness that dolutegravir has interactions	*n* = 1636^a^	*n* = 1950
Yes	1365 (83.4)	1365 (70.0)
No	271 (16.6)	271 (13.9)
Missing	0 (0)	314 (16.1)
Awareness of specific dolutegravir interactions	*n* = 1333^b^	*n* = 1636^c^
Calcium	820 (61.5)	820 (50.1)
Iron	714 (53.6)	714 (43.6)
Magnesium/aluminium	713 (53.5)	713 (43.6)
Rifampicin	1158 (86.9)	1158 (70.8)
Metformin	1042 (78.2)	1042 (63.7)
Carbamazepine	783 (58.7)	783 (47.9)
Phenobarbitone	592 (44.4)	592 (36.2)
Phenytoin	668 (50.1)	668 (40.8)
Non‐interacting medicines		
Oral contraceptives	234 (17.6)	234 (14.3)
Lamotrigine	181 (13.6)	181 (11.1)
Sodium valproate	349 (26.2)	349 (21.3)

^a^ This denominator excludes those who had dropped out by/in this section (314).

^b^ The survey was designed using branching logic. Those who answered that they were not aware that dolutegravir has interactions did not see this question and those who dropped out (missing) were excluded.

^c^ This denominator includes those who were unaware of interactions but excludes those who had dropped out by/in this section.

**Figure 1:**
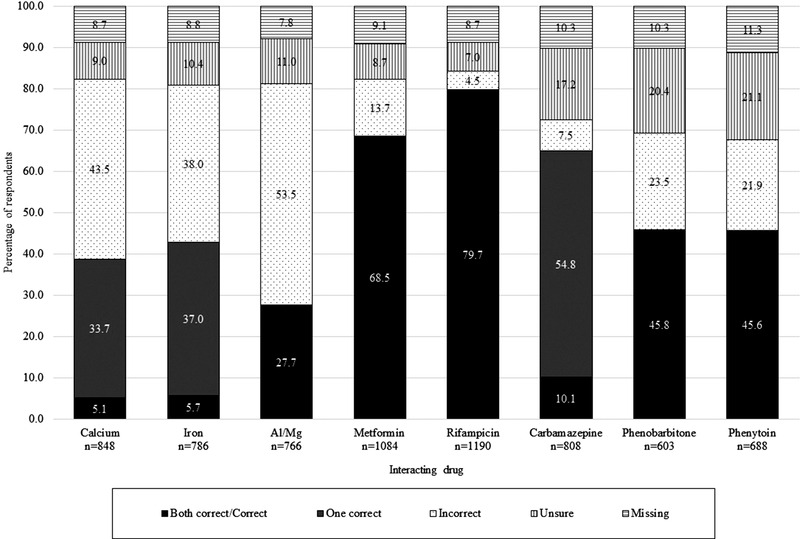
Knowledge of specific dosing changes needed due to dolutegravir interactions.

Knowledge of how to adjust dosing was poor, except with rifampicin and metformin (Figure 1). It must be noted that the proportions did not include all respondents, so while it is reassuring that 86.9% of the respondents were aware of the interaction with rifampicin (Table 2) and 79.7% of those knew to double the dose of dolutegravir (Figure 1), the branching nature of the survey excluded those unaware of interactions at all (13.9%) and those who did not respond to this question (16.1%), so these proportions may over‐estimate knowledge. As an example, when including all respondents who saw the interaction section, 70.8% were aware of the rifampicin interaction (Table 2). This is especially concerning in a country with a TB incidence of 357/100,000 in people living with HIV [38].

When asked to pick the correct dosage regimen to counteract the interaction between cation‐containing medicines and dolutegravir (with food or 2 hours after/6 hours before dolutegravir), 5.1% (calcium) and 5.7% (iron) picked both correct options, and 33.7% (calcium) and 37.0% (iron) picked one of the two (Figure 1). The significant gap in the knowledge of interactions with cation‐containing medicines is a major concern, especially in pregnant women, who routinely take calcium and/or iron and antacids. Serious repercussions on maternal health and the risk of mother‐to‐child transmission may result.

With the anti‐epileptics, 10.1% chose both correct options for carbamazepine and 54.8% chose one of the two correct options. The contraindication to using phenobarbitone and phenytoin in patients on dolutegravir was known by 45.8% and 45.6% of HCWs, respectively (Figure 1).

### 3.3 Variables influencing dolutegravir interaction knowledge

HCWs with guideline access were more likely to be aware of interactions (90.5%, 95% CI 89–92 vs. 61.6%, 95% CI 57–66) and have dosing knowledge (rifampicin: 91.4%, 95% CI 89–93 vs. 66.5%, 95% CI 59–73). Similarly, training was positively associated with interaction awareness (96.6%, 95% CI 95–98 vs. 61.9%, 95% CI 58–66) and dosing knowledge (rifampicin: 92.5%, 95% CI 91–94 vs. 72.7%, 95% CI 67–78). With just over half of our respondents (56.6%) having received training (Table 1), this is a key point for intervention. When looking at training by profession, 55.7% of doctors reported having received training, 62.1% of nurses and 50.9% of pharmacists (Table 1). A study of primary care nurses by Kredo et al. found that patchy and non‐inclusive training on guidelines was a major barrier to their use [39]. The need for widespread training on new guidelines is clear.

Nurses were less likely than doctors to be aware that dolutegravir has interactions (82.5%, 95% CI 80–85 vs. 90.7%, 95% CI 88–93). Nurse knowledge of dosing changes required revealed that 31.5% knew that of magnesium/aluminium, 86.1% rifampicin, 68.2% metformin and 8.5% carbamazepine. The huge HIV burden in South Africa and staff constraints, where doctors and pharmacists may visit rural facilities infrequently, places nurses at the frontline of HIV care. It is imperative that nurses are fully versed in dolutegravir's interactions and the necessary dosing adjustments.

### 3.4 Strengths and limitations

The primary strength of this study was the good uptake and broad access to HCWs across South Africa, allowing the results to be generalizable. Our study had several limitations. The survey was sent out between the first and second waves of the COVID‐19 pandemic in South Africa, so training and guideline access may have been negatively influenced by “rolling” countrywide lockdowns, strained healthcare resources and physical distancing.

Our study had the potential for selection and non‐response bias. Dissemination to users of the hotline and HIV‐centred organizations meant that we were targeting the “interactive” group, that is HCWs who actively seek information. Similarly, the survey was targeted at HCWs working in the field of HIV, which may have resulted in over‐reporting. In a country with an HIV incidence of 3.98/1000 in 2019 [1], antiretrovirals may routinely be prescribed and dispensed by HCWs not specifically working in the HIV sphere.

## 4 CONCLUSIONS

Our study has revealed gaps in the awareness and knowledge of dolutegravir interactions and how to adjust dosing among South African HCWs in the field of HIV. This study highlights the need to train more HCWs, especially nurses, who are at the forefront of HIV care in South Africa. The development of novel, inexpensive and accessible training methods, for all professions, especially in the context of COVID‐19 and in countries with large rural populations and facilities, is vital.

## COMPETING INTERESTS

The authors declare no competing interests.

## AUTHORS’ CONTRIBUTIONS

BSC, AMS and MB conceived the research project. BSC designed the survey with input from AMS and MB. The survey was tested, piloted, disseminated and conducted by BSC. Data were extracted by BSC, cleaned by BSC, in consultation with AMS and MB, and analysed by BSC and statisticians. The first draft of the manuscript was written by BSC and revised by BSC, AMS and MB. All authors have read and approved the final manuscript.

## FUNDING

This research was made possible under funding provided by the Global Fund to Fight AIDS, Tuberculosis and Malaria through the National Department of Health of South Africa and the NDoH Pharmacovigilance Centre for Public Health Programmes.

## DISCLAIMER

This research, the results and discussion are solely the responsibility of the authors and do not necessarily represent the official views of the Global Fund or the National Department of Health of South Africa.

## Data Availability

The data that support the findings of this study are available from the corresponding author upon reasonable request.
